# The Role of Different Sugars, Amino Acids and Few Other Substances in Chemotaxis Directed Motility of *Helicobacter Pylori*

**Published:** 2012

**Authors:** Hamid Abdollahi, Omid Tadjrobehkar

**Affiliations:** 1 *Microbiology Department, Medical School, Kerman University of Medical Sciences, Kerman, Iran*; 2 *Microbiology Department, Medical School, Kerman University of Medical Sciences, Kerman, Iran and Basic Sciences Department, Medical School , Zabol University of Medical Sciences, Zabol , Iran*

**Keywords:** Amino acids, Bicarbonates, Chemotaxis, Helicobacter pylori, Urea

## Abstract

**Objective(s):**

Motility plays a major role in pathogenicity of *Helicobacter pylori*, yet there is scarce data regarding its chemotactic behaviour. The present study was designed to investigate the chemotactic responses of local isolates of *H. pylori *towards various sugars, amino acids, as well as some other chemical substances.

**Materials and Methods:**

Chemotaxis was assayed by a modified Adler’s method. We used solutions of sugars, amino acids as well as urea, sodium chloride, sodium and potassium bicarbonate, sodium deoxycholate and keratin at 10 mM concentrations.

**Results:**

Despite some small differences, tested *H. pylori* isolates generally had a positive chemotaxis towards the tested sugars (*P*< 0.05). Among amino acids, phenylalanine, aspartic acid, glutamic acid, isoleucine and leucine showed a positive chemotaxis (*P*< 0.05) ; however, tyrosine showed negative chemotaxis (repellent) (*P*< 0.15). Urea, sodium chloride, sodium and potassium bicarbonate showed to be attractants (*P*< 0.05), but sodium deoxycholate was repellent (*P*< 0.05).

**Conclusion:**

It seems that, sugars and many amino acids by their attraction for *H.pylori*, many amino acids, may enhance the activity of this bacterium and probably aggravate the symptoms of its infection. However, those like L-tyrosine, may possibly be employed as deterrents for *H. pylori *and thus can control its infections. However, we suggest that further investigations on chemotactic behaviour of many more strains of *H. pylori *should be carried out before a final conclusion.

## Introduction

*Helicobacter pylori* is a human gastric pathogen that is associated with the development of different diseases ranging from gastritis, duodenal and gastric ulcers, to gastric adenocarcinoma (1).

Motility is an important virulence factor for *H. pylori*. This characteristic may facilitate colonization of gastric mucosa by* H. pylori*, as non-motile or weakly motile organisms which are avirulent or less virulent (2-5)*.* Bacterial motility is entwined with chemotaxis (6, 7)*.* Most flagellated bacteria, perform chemotactic motility by the recognition of environmental conditions, such as solute concentration, coupled with regulation of swimming behaviour (8, 5)*.*

Nakamura *et* *al *(9) have demonstrated the role of urease activity in motility of *H. pylori *in a viscous environment, and concluded that cytoplasmic urease plays an important role in the chemotactic motility of this bacterium. Mizote *et* *al* (10), however, showed that *H. pylori *can have chemotactic responses to urea, urease inhibitors and sodium bicarbonate independent from urease activity. These workers justified that chemotactic behaviour of this organism towards urea and sodium bicarbonate is essential for colonization and persistence of this organism in the stomach.

Chemotaxis directed motility is a critical characteristic in growth cycle of *H. pylori *in gastric mucosal layer. Terry *et al* (11) have indicated that chemotaxy is an essential element in establishing and maintaining infection in all regions of stomach. It can be postulated that, successfull colonization of gastric mucosa probably occurred when chemotactically active *H. pylori *were attracted to deep gastric lumen by certain chemical stimulus and proliferated on epithelial cell surface. Bacteria, then leave condition towards more variable pH region and repeat the cycle when return again to the vicinity of the gastric mucosal layer cells by repultion of acidic pH (12)*.* This cycle is directly affected by different chemical substances, continuously secreted from gastric lumen and decreased concentration by a sharp gradient from deep luminal to epithelial surface (13)*.* Therefore, attraction or repultion of chemical component of nutrients could be another responsible factor in growth cycle of *H. pylori *in gastric mucosal layer. This study was designed to investigate the role of a wide range of sugars and amino acids in attracting or repelling local isolates of *H. pylori*, and to compare them with few other studies in other parts of the world. It was hoped that chemotactic data for *H. pylori *be might of some use in controling or preventing its infection by means of food diets with regard to their contents.

## Materials and Methodes

***Bacterial strains and growth conditions***

In this study, three bacterial isolates which were cultured from gastric biopsies, taken from symptomatic patients referred to gastroscopy unit at Kerman University Hospital, were used. These isolates, were cultured in brucella broth, supplemented with 7% horse serum and incubated in candle jars at 37 ºC for 3-5 days. Grown bacteria were sub-cultured in the same medium with 20% glycerol, and then refrigerated at -20 ºC till their use.

Bacterial assesment for urease, motility, oxidase and catalase activity were performed before chemotaxis assay to confirm existence of viable and active bacteria and absence of contaminations. Urease production was assesed by inoculation to Christensen's urea agar and we confirmed the motility of isolates by phase contrast microscopy.

***Chemicals***

All of the chemicals (urea, sodium chloride, sodium and potassium bicarbonate, sodium deoxycholate and keratin), sugars (D-fructose, D-lactose, D-glucose, D-mannose, D-xylose, D-mannitol, D-sorbitol, D-arabinose, D-maltose, D-sucrose) and amino acids (L-serine, L-alanine, L- tyrosine, L-aspargine, L-phenylalanine, L-tryptophane, L-leucine, L-proline, L-valine, L-aspartic acid, L-glycine, L- isoleucine, L- cysteine, L-glutamic acid, L-histidine, L-lysine) were obtained from Merck . These chemicals were used as 10 mM solutions in chemotaxis assay.

***Chemotaxis buffer***

Ten mM potassium phosphate buffer solution at pH 7.0 was used as chemotaxis buffer and washing solution for rinsing outer walls of the capillary tubes.

***Chemotaxis assay***

Chemotaxis was assayed by a modified method of that described by Adler (6)*.* In brief; a small chamber was made from a V-shape sealed capillary tube glued on a glass slide, was covered with a cover slip, and then was filled with 200 µl of washed bacterial cell suspension in chemotaxis buffer adjusted to a concentration of about 3 x 10^8^ cells per ml . The chemotaxis buffer was also used for washing cells twice by centrifugation at *g* for 10 min at 4 ºC. Then 20 µl capillary tubes were first sealed at one end by flame and then quickly passed over Bunsen flame and inserted into the 10 mM solutions of tested chemicals which had been dissolved in chemotaxis buffer. These were incubated for ten minutes, then after rinsing the outer walls of capillary tubes, they were inserted slowly into the chemotaxis chamber which contained the bacterial cell suspension. After incubation at room temperature for 60 min, capillary tubes were removed from the chamber, the sealed end of tubes were gently broken and the tube incorporated bacteria were spread over a known area on a glass slide, Gram stained, and counted in 10 random microscopic fields (bright field microscope). Chemotaxis assays were applied for three different isolates and repeated at least four times for each isolate.

Our results are expressed as the actual bacterial cell concentration per ml, for each case the number of the cells entered into the control tubes was deducted from that of tubes containing tested substrates and the control was regarded as zero. Depending on the mean number of cells, chemotactic responses were considered as positive, negative or inert when the relevant cell numbers were above, below or equal to zero respectively.

***Statistical analyses***

The significance of differences between the mean results obtained from different chemical substances and PBS as control were analyzed by one sample t-test. For most cases *P*< 0.05 was considered statistically significant, but for few certain cases where the differences were likely *P*< 0.15 were also used.

## Results

The three bacterial isolates used in all of the chemotaxis assesments showed some small variations in chemotactic responses, which are shown as standard deviation for the mean cell numbers in the relevant cases.

***Chemotactic responses to sugars***

**Figure 1:**
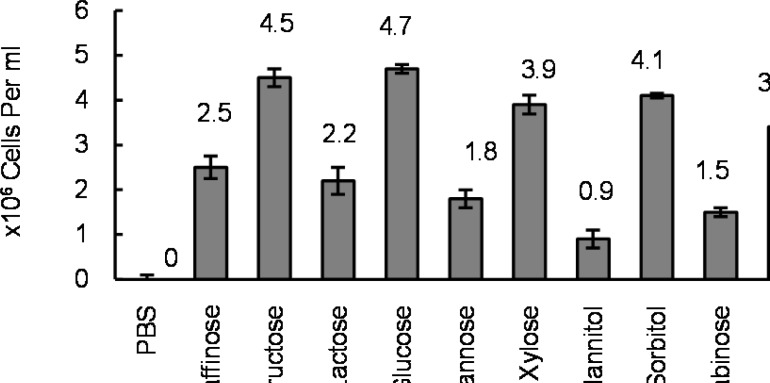
Bacterial cell concentration (Mean number ± SD) entered the capillary tube containing different sugars at 10 mM concentrations

Results revealed that all of the tested sugars except manitol were significant chemoattractants (*P*< 0.05). Glucose and fructose seemed to be the strongest but arabinose the weakest of attractants for the tested isolates (Figure 1). 

***Chemotactic responses to amino acids***

**Figure 2:**
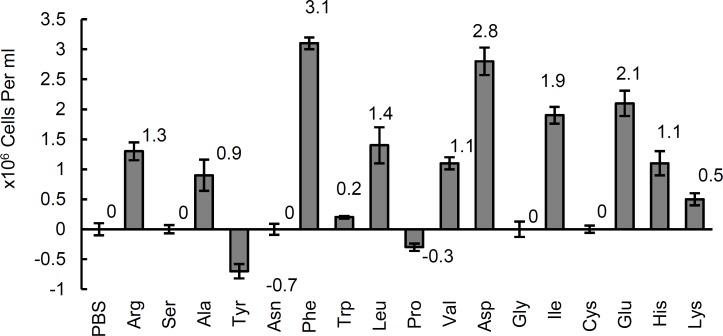
Bacterial cell concentration (Mean number±SD) entered the capillary tube containing different amino acids at 10 mM concentrations.

Among the 17 amino acids tested here; phenylalanine, aspartic acid, glutamic acid, isoleucine and leucine showed a positive chemotaxis, phenylalanine had the strongest attraction (*P*< 0.05). Tyrosine showed a negative chemotaxis (*P*< 0.15) as can be seen in Figure 2. Despite some apparent differences between certain amino acids and the control (Figure 2), there were no statistically significant differences (*P*< 0.15). On the basis of our results, there were statistically significant differences among some of the tested amino acids and control with 85% confidence interval, but these differences were not significant at 95% confidence interval (Table 1).

**Table 1:** Chemotactic responses of *Helicobacter* *pylori *to different substances expressed as bacterial cell concentration per ml ± SD entered the capillary tube containing different substances at 10 mM concentrations ( control values were deducted from them)

Substrate	10^6^	*P -value
Urea	3.9 ± 0.23	0.001
Keratin	- 0.3 ± 0.05	0.449
Potassium bicarbonate	4 ± 0.15	0.001
Sodium bicarbonate	3.8 ± 0.3	0.001
Sodium chloride	2.8 ± 0.1	0.005
Sodium deoxycholate	- 1.3 ± 0.08	0.001
Sugars		
D-Raffinose	2.5 ± 0.25	0.007
D-Fructose	4.5 ± 0.2	0.001
D-Lactose	2.2 ± 0.3	0.012
D-Glucose	4.7 ± 0.1	0.000
D-Mannose	1.8 ± 0.2	0.026
D-Xylose	3.9 ± 0.21	0.001
D-Mannitol	0.9 ± 0.2	0.180
D-Sorbitol	4.1± 0.05	0.001
D-Arabinose	1.5 ± 0.1	0.048
D-Maltose	3.4 ± 0.3	0.002
D-Sucrose	2.6 ± 0.08	0.006
Amino acids		
L-Arginine	1.3 ± 0.15	0.074
L-Serine	0 ± 0.07	0.700
L-Alanine	0.9 ± 0.26	0.181
L- tyrosine	- 0.7± 0.12	0.113
L-Aspargine	0 ± 0.09	0.610
L-Phenylalanine	3.1± 0.1	0.003
L-Tryptophane	0.2 ± 0.02	0.607
L-Leucine	1.4 ± 0.3	0.060
L-Proline	- 0.3 ± 0.06	0.449
L-Valine	1.1 ± 0.1	0.115
L-Aspartic acid	2.8 ± 0.23	0.005
L-Glycine	0 ± 0.13	0.570
L- Isoleucine	1.9± 0.14	0.022
L- Cysteine	0 ± 0.06	0.432
L-Glutamic acid	2.1± 0.21	0.015
L-Histidine	1.1± 0.2	0.115
L-Lysine	0.5 ± 0.1	0.229

*One sample t-test compared means with control. NS, not significant difference at 95% confidence interval

NS†, no significant difference at 95% confidence interval but significant at 85%confidence interval

***Chemotactic responses to other chemicals***

**Figure 3:**
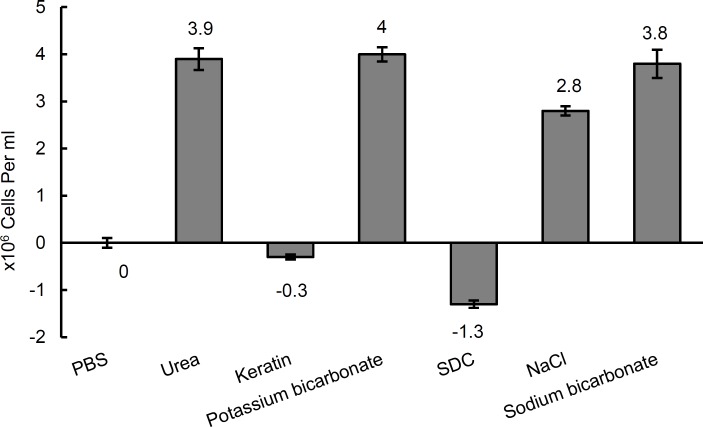
Bacterial cell concentration (Mean number±SD) entered the capillary tube containing different substances at 10 mM concentration

Results from this study showed that, urea, sodium chloride, sodium and potassium bicarbonate induced positive chemotactic responses (*P*< 0.05), while sodium deoxycholate showed a potent negative chemotaxis (*P* < 0.05) and it seems that keratin do not have any appreciable effect on *H. pylori *(Figure 3)*.*

## Discussion

Motility is regarded as a major virulence factor for* H. pylori *and it has been shown that non-motile organisms are avirulent or less virulent (4, 11, 14, 15)*.* It is therefore reasonable to assume that those chemicals, which are capable of inducing motility, may play a role in virulence or affecting the severity of symptoms of *H. pylori* infections.

According to the results of this study, all tested sugars except mannitol showed a positive chemotaxis (Figure 1), and this may mean that food diets with high sugar contents could facilitate the motility of *H. pylori* or aggravate the symptoms of its infection. This may particulary be substantial in the case of glucose, which showed to be the strongest attractant as well as being a carbon-energy source for *H. pylori* growth (16). 

There are also many supporting reports that indicate the effect of various virulence factors such as VacA in a rapid increase in ion conductivity and consequently, enhancing ions and small neutral molecules such as sugars trans-epithelial diffusion and increases the supply of essential nutrients that are necessary for bacterial growth on the mucosa (17).

There is considerable diversity in amino acids requirement of different *H. pylori *strains, but in general, most of the strains have an absolute requirement for 7-8 amino acids including arginine, histidine, isoleucine, leucine, methionine, phenylalanine and all of the strains require valine. Some amino acids such as tyrosine, cysteine and glycine are considered non-essential (18, 19)*.*

Our results showed that some amino acids like phenylalanine, aspartic acid, glutamic acid, isoleucine and leucine were chemoattractant whereas others were mainly indifferent at 95% confidence interval. However, at 85% confidence interval arginine, valine and histidine were also regarded as chemoattractant but tyrosine behaved as a repellent (Table 1).

Interestingly enough our data showed that chemotaxis responses to amino acids are in accordance with amino acids requirement of *H. pylori*. We examined all of the *H. pylori*’s essential amino acids except methionine which was not available in our experiments. Most of the tested amino acids behaved as chemoattractant.

A few amino acids like aspartate and glutamate are normally used by* H. pylori *to facilitate the production of ammonia (20) for protection against highly acidic environment of the stomach and increasing adherence to gastric epithelial cells during the initial stages of colonization (21, 22)*.* Regarding the report which showed that arginine, aspartate, glutamate and serine play a role as carbon-energy source for *H. pylori *(20), it is reasonable to find such amino acids as attractants and we did so in our study. Nevertheless, the high diversity among the isolates of *H. pylori *on the basis of amino acids requirements should not be forgotten as reported in previous studies (18)*.*

Our results showed a significant positive chemotaxis to glutamic acid and aspartic acid (Figure 2), which contradicts with Worku *et al* (23) observations, which showed glutamate and aspartate as repellents. This may be due to variations in characteristics of the isolates used in the two studies.

Most of the amino acids which showed positive chemotaxis in this study, correspond with those required as growth factors, but, glutamic acid, despite its positive chemotaxis is not considered as a growth factor (Figure 2). Whether this controversy is due to the differences of bacterial strains used here and in the study of Reynolds and Penn (19)*,* or in fact chemotaxis and growth requirements should be regarded as separate entities of bacterial cells, is questionable.

Among 17 tested amino acids, L-tyrosine was a distinct repellent (Figure 2). Hypothetically, consumption of this amino acid as a food additive may decrease colonization of *H. pylori,* but whether this amino acid could actually play a role in preventing or reducing the symptoms of *H. pylori’s* infections remains to be investigated. 

 The chemoattractant amino acids were generally weaker attractants than the sugars, and L-tyrosine was a repellent. The actual role of each amino acid in preventing or inducing the activity of *H. pylori *and consequently decreasing or aggravating the symptoms of its infections remains to be seen in further investigations. 

The repulsion of *H. pylori *against deoxycholate in this study corresponds well with the study of Graham and Osato (24)*.* The unsuccessful colonization of lower parts of digestive system by *H. pylori *may partly be due to the excretion of deoxycholate from gall bladder into the intestine, though the lack of favorable environmental conditions such as urea deficiency or other essential substrates in these parts should also be taken into account. The attraction of urea, potassium and sodium bicarbonate as well as sodium chloride for *H. pylori *in this study (Figure 3) correlate well with the findings of previous studies (10, 25, 26), and this may prove the validity of the methodology used here.

## Conclusion

It has been shown that nutrients released by gastric epithelium, enhance the growth of *H. pylori* (27)*,* yet the low prevalence of its infection in some parts of the world has been attributed to the certain food diets (28)*.* Therefore we suggest further similar investigations to be carried out with *H. pylori *strains isolated in different parts of the world, and a collective data analysis should determine the role of various substances as attractants or repellents. Such data could then be implied as a guideline in food diets or medicinal drugs to control the *H. pylori *infections. If accumulative data in this area became available, we maybe able to control at least the symptoms of *H. pylori* infections by means of food diets in a much better way than the vague diets being used at present.
